# Supplementary material for: Quantitative interactome mapping of skeletal muscle insulin resistance
Source: Mol Syst Biol. 2026 Jun 23;22(9):1430–52. doi: 10.1038/s44320-026-00224-7 (PMC13538405; doi:10.1038/s44320-026-00224-7)
Supplement: Supplementary file 1 [file 44320_2026_224_MOESM1_ESM.pdf]

Quantitative interactome mapping of skeletal muscle insulin resistance

Yaan-Kit Ng, Ronnie Blazev, Julian Wong, Nichollas Scott, Jeffery Molendijk, and Benjamin Parker

Corresponding author(s): Benjamin Parker (ben.parker@unimelb.edu.au)

Review Timeline:

Transfer from Review Commons:	7th Apr 26
Editorial Decision:	15th Apr 26
Revision Received:	1st May 26
Editorial Decision:	28th May 26
Revision Received:	2nd Jun 26
Accepted:	9th Jun 26

Editor: Poonam Bheda

Transaction Report: This manuscript was transferred to Molecular Systems Biology following peer review at Review Commons.

Review #1

1. Evidence, reproducibility and clarity:

Evidence, reproducibility and clarity (Required)

Summary

Ng et al. use a combination of quantitative (structural) proteomics tools to study the effect of insulin resistance (IR) on protein interactions in mouse muscle cells (C2C12 myotubes) and muscle tissue. First, the authors use protein correlation profiling (PCP; co-fractionation) to separate protein complexes from C2C12 cells with and without induced insulin resistance. The PCP data is then used to reconstruct protein complexes and networks based on binary interactions and to uncover changes upon IR, this is illustrated by several examples (Fig. 1). As an orthogonal method quantitative cross-linking (XL) is used to study protein interactions in C2C12 cells. A multidimensional enrichment/fractionation scheme is designed to increase the number of cross-links/protein-protein interactions (PPIs), and again interaction networks are generated and changes between no IR vs. IR conditions assessed (Fig. 2). The XL approach is then carried forth into a mouse model of IR. Muscle tissue of mice fed on regular chow or on a high-fat diet are compared (Fig. 3). Selected XL data is validated on known structures and quantitative data from PCP and XL is compared to integrate the different interactome data (Fig. 4). Finally, the interactions of PDIA6, a key protein found to be affected by IR, are studied in more detail in the mouse model. The effect of overexpression of PDIA6 is studied using different readouts, including redox proteomics (Fig. 5). These data connect the redox imbalance / cysteine oxidation with the role of PDIA6 in insulin resistance.

Overall, the manuscript is impressive with respect to the methodological effort undertaken, generating and combining many large-scale proteomics data sets, both from a cell line and from mouse tissue. The large amount of data accumulated can be seen as a strength and as a weakness, because it is impossible to follow up on all findings (changes of interactions induced by IR observed in any sample and with any method). Nevertheless, the PDIA6 example was evaluated in more detail and with dedicated follow-up experiments. The conclusions from this experiment are plausible and presented logically. It is, however, difficult for a reviewer to quickly judge whether there would have been more promising leads for validation experiments than PDIA6.

Given the large amount of data already generated, I do not have suggestions for additional experiments. Instead, some analysis and interpretation of existing data need further

clarification.

Major comments

The search strategy and statistical treatment of the cross-linking data need to be explained more clearly. The authors write that the data were searched with pLink2 and the FDR was controlled at 1%. However, it remains unclear whether the FDR was controlled at the level of peptide/cross-link-spectral-matches (PSMs/CSMs), or at the level of non-redundant site pairs or even protein-protein interactions for inter-protein links, which would be more appropriate. Controlling the FDR only at the PSM/CSM level will lead to an inflation of false positives when aggregating results at the interaction level. Moreover, it remains unclear whether all data sets were searched together or whether individual data sets or subsets of the entire data were searched individually. For example, if single files or only files belonging to a certain higher-order fraction would be searched individually, this would again lead to an underestimation of false positives. What is particularly noteworthy in this context is that in a typical dataset inter-protein links are underrepresented compared to intra-protein links for statistical reasons, while the numbers in this manuscript are much more balanced (almost equal numbers in the cell line data set, and even more inter-protein links than intra-protein links in the tissue). The authors should consider articles that discuss FDR control in XL-MS, for example by the Rappsilber group. Minimally, more details about what was searched together and at what level the FDR was controlled need to be provided.

On a similar note, it has been discussed in the literature that validating large-scale XL data on selected structures of complexes is a poor proxy for accurate FDR control, as such complexes are commonly not representative for more transient or substoichiometric PPIs, or PPIs involving low-abundant proteins.

Minor comments

Figure 1: CCT is a complex composed of eight subunits, but only seven are shown. What happened to the remaining one (CCT6)?

The authors performed a redox proteomics experiment in a PDIA6 overexpression system. However, the statement in the Conclusion section that PDIA6 overexpression promotes disulfide bond formation in interacting proteins is not directly justified because the method only quantifies cysteine oxidation, not S-S bond formation directly.

All supplementary data is not provided in an independent repository, but in a repository of the authors' institution. It is unclear whether the data could be accessed anonymously. Proteomics data need to be provided in an independent, community accepted repository such as from the members of the proteomeXchange consortium (PRIDE etc.).

A clear description of what is shown in the SI tables is missing, e.g. in the form of figure legends. In their present form, SI data are difficult to interpret. For example, I did not find information about cross-link identifications, only quantitative data on cross-link changes. However, if the identification of a cross-link is not confident in the first place (see my comments above), then the quantification will be irrelevant.

Referees cross-commenting

I trust the expertise of reviewer #2 on matters related to insulin resistance. It seems that we both agree that the PDIA6 example might require a more consistent justification throughout the manuscript.

2. Significance:

Significance (Required)

The study is one of only a few so far that combines PCP and XL on such a large scale for a mammalian system. There are also very few studies of cross-linking on tissue. Therefore, from a methodological point of view, the study is highly innovative. The application to the muscle cell system and insulin resistance as a biological research question is furthermore very novel. As such, the study is valuable to different communities - those developing and refining experimental methods and those using them to uncover regulatory mechanisms. Another strength is that the authors made serious efforts at each step to optimize the XL method and adapt it to their sample types of interest.

The wealth of data is both a strength and a weakness of the work. Inevitably, a reviewer might argue that some aspects of the work could have been done differently. Unless someone spends a lot of time going deep into the result tables, it will be difficult to make constructive suggestions on additional targets for further investigation. Nevertheless, some statistical aspects of data analysis need to be clarified, and parts of the data analysis might need to be repeated. This, in turn, may require some reinterpretation of findings related to the XL data.

Advances: Conceptual, methodological, mechanistic

Audience: Specialized, basic research, translational

Reviewer expertise

My background is in proteomics, structural proteomics, mass spectrometry, analytical sciences, experimental methodology, and computational data analysis. I have general knowledge of biological processes, but I am not an expert on insulin resistance.

3. How much time do you estimate the authors will need to complete the suggested revisions:

Estimated time to Complete Revisions (Required)

(Decision Recommendation)

Between 1 and 3 months

4. *Review Commons* values the work of reviewers and encourages them to get credit for their work. Select 'Yes' below to register your reviewing activity at Web of Science Reviewer Recognition Service (formerly Publons); note that the content of your review will not be visible on Web of Science.

Web of Science Reviewer Recognition

Yes

Review #2

1. Evidence, reproducibility and clarity:

Evidence, reproducibility and clarity (Required)

Quantitative interactome mapping of skeletal muscle insulin resistance
Ng et al present a series of proteomics/interactomics studies in skeletal muscle to identity insulin regulated complexes/interactions and changes ot these in insulin resistant muscle. More mechanistically, the Authors focus on changes in interactions involving chaperones

in the ER/SR, presenting interesting data on the effect of PDIA6 overexpression alters insulin sensitivity in muscle ex vivo.

Major Comments:

The section entitled "Validating the regulation of PPIs with insulin resistance in C2C12 myotubes with quantitative XL-MS". This is not really a validation of th previous data as presented, but more an orthologous assay that helped pinpoint the interest in the ER. Suggest adjusting the title.

Figure 3B - the "decrease" in AS160 pS588 regulation appears to be due to increased basal, not decreased phosphorylation in after insulin. This should be commented on or clarified.

PDIA6 is down-regulated in muscle from people with T2D - so why did the authors decide to overexpress PDIA6? I note this rationale is explained in the discussion, and could be articulated better in the results.

Figure 5J and K. The TA muscles are substantially larger from PDIA6 OE mice. Are the muscle fibres also larger? Tbhis relates to the normalisation of data in K. This appears to be normalised to g tissue. If so, is the difference between control, and OE mice being driven by the increase in muscle mass - with uptake per muscle or per fibre the same?

Minor Comments:

For the PCP-MS data form C2C12 cells. The authors use an analysis of AUC to assess protein abundance, which, as they state, is important for chronic treatments if total protein is not separately quantified. However, the analysis of changes in protein distribution is less clear from the text in the results section. Intuitively, a profile that is normalised to total intensity in all fractions would provide a protein abundance-independent read-out for changes in protein distribution. Does the "local analysis" capture this same information? Could the Authors provide a little more information here?

Figure 1M - are the Authors sure that VPS41 should be in this panel. It doesn't seem to be insulin regulated, and the arrow appears to refer to movement between insulin sensitive and insulin resistant.

Figure 1N - "This includes an array of TBC1 domain-containing proteins (TBC1D15, 195 TBC1D17, TBC1D8B) that are consistently reduced with IR". Do the Authors mean the

abundance was less, or that complex formation was reduced?

Optional. In general, there is a lot of text discussing the literature around proteins highlighted in the analysis. This is useful to an extent, but the Authors might consider streamlining this a little (perhaps moving some of the information ot supp tables?).

Why do the Authors think the crosslinking MS was not able to capture acute PPI changes like the PCP-MS was?

For the EDL crosslinking data. Are the Authors able to provide a comparison with C2C12 data - to highlight the differences and similarities between tissue and the cell model? This may be a challenge if the authors think most differences may be technical.

Please check - "reduces free-glycerol levels essential for fatty acid synthesis". Glycerol does not directly contribute to FA synthesis. But is needed for triglyceride synthesis.

Do the Authors think that the change in PDIA6 interactions may be a general/indirect indication of changes in ER redox and/or protein misfolding in insulin resistance?

Is PDIA6 an ER luminal protein? If so, it being phosphorylated is interesting.

Referees cross-commenting

Similarly, reviewer #1 raises important points on the description of key parts of the analysis, that will need to be addressed. I think we agree that the manuscript emcpmpasses a great deal of data, and that it is somewhat difficult to follow why PDIA6 was selected for validation. Overall, the reviews pick up on different aspects of the manuscript that could be improved.

2. Significance:

Significance (Required)

Overall, the strength of the paper is in the underlaying proteomics workflows and analysis. The work presented of very high technical quality, and I have no doubt the data presented will be of use to the field beyond the analysis in this current publication.

However, a weakness is doubts over the relevance of the data on PDIA6 overexpression in

muscle insulin resistance.

This will be of interest to those in the proteomics, interactomics and metabolism fields.

My expertise is in glucose metabolism, insulin signalling and insulin resistance.

3. How much time do you estimate the authors will need to complete the suggested revisions:

Estimated time to Complete Revisions (Required)

(Decision Recommendation)

Less than 1 month

4. *Review Commons* values the work of reviewers and encourages them to get credit for their work. Select 'Yes' below to register your reviewing activity at Web of Science Reviewer Recognition Service (formerly Publons); note that the content of your review will not be visible on Web of Science.

Web of Science Reviewer Recognition

Yes

Revision Plan

Manuscript number: RC-2026-03377
Corresponding author(s): Benjamin L. Parker

1. General Statements

We are delighted that both reviewers agree our manuscript is "impressive with respect to the methodological effort undertaken, generating and combining many large-scale proteomics data sets, both from a cell line and from mouse tissue" and "the study is highly innovative" (Reviewer 1), and "the work presented of very high technical quality" and "will be of interest to those in the proteomics, interactomics and metabolism fields" (Reviewer 2).

We are also encouraged that there are no major concerns with regards to experimental design and no additional experiments have been suggested.

2. Description of the planned revisions

Reviewer 1: PSM/CSM vs non-redundant site or inter-protein link False discovery rate (FDR).
We will process our data with xiFDR from the Rappsilber Laboratory as suggested by the Reviewer which is compatible with our current output from pLink2 to investigate how this influences FDR. We will modify or provide additional figures with these analysis if required. We will provide more information on how the batches of data were searched / processed.

Reviewer 1 & 2: Validating PPI's with XL-MS or *in silico* analysis on 3D structures.
We will change the phrasing and discussion from 'validating' to orthologous analysis throughout the manuscript.

Reviewer 1 & 2: Justification for analysis of PDIA6
We will provide a greater justification and rationale for studying PDIA6 in more detail and revise other minor points/questions relating to PDIA6 interactions.

Reviewer 1: Supplementary Data.
We have already started to upload all data to PRIDE ProteomeXchange as suggested by the reviewer (see below). We will provide clearer descriptions of the current Supplementary Tables.

Reviewer 1 & 2: Minor comments throughout.
We will edit various minor comments as suggested by the Reviewers.

3. Description of the revisions that have already been incorporated in the transferred manuscript

We have not made any text changes to the manuscript at this stage. However, we have already uploaded two out of the four datasets to PRIDE:

Project Name: PCP-MS analysis of insulin resistance in C2C12 myotubes with or without acute insulin stimulation
Project accession: PXD075147
Token: F2PAyNGyAhOx
Alternatively, reviewer can access the dataset by logging in to the PRIDE website using the following account details:
Username: reviewer_pxd075147@ebi.ac.uk
Password: uGiWJuTbfrFd

Revision Plan

Project Name: Cysteine Redox proteomics of PDIA6 over-expressing skeletal muscle
Project accession: PXD075204
Token: 0n6Zyyhn8lGi
Alternatively, reviewer can access the dataset by logging in to the PRIDE website using the following account details:
Username: reviewer_pxd075204@ebi.ac.uk
Password: ejkGBy3ALCxF

4. Description of analyses that authors prefer not to carry out

Please include a point-by-point response explaining why some of the requested data or additional analyses might not be necessary or cannot be provided within the scope of a revision. This can be due to time or resource limitations or in case of disagreement about the necessity of such additional data given the scope of the study. Please leave empty if not applicable.

Both reviewers comments are great and we are confident the proposed revisions will improve the manuscript. We have no issues with any comment.

It is worth noting that processing .raw proteome-wide XL-MS data is computationally demanding and took >1 month even on a computer with 128 cores and 1TB of RAM. Therefore, we have prioritised post-processing these searched data to assess FDR. Re-searching the .raw data with another XL-MS software package is not feasible.

15th Apr 2026

Manuscript Number: MSB-2026-13739-T
Title: Quantitative interactome mapping of skeletal muscle insulin resistance

Dear Dr. Parker,

Thank you again for submitting your work to Molecular Systems Biology. We have now discussed your manuscript, the reviews from Review Commons, and your revision plan to address the remaining reviewer comments. We are happy to let you know that with the addition of the proposed revisions, we would send your paper back to the original reviewers who reviewed your paper at Review Commons for re-review at Molecular Systems Biology. Please let me know in case you would like to discuss in further detail, I would be happy to schedule a call.

When submitting your revised manuscript, please carefully review the instructions that follow below. We perform an initial quality control of all revised manuscripts before re-review; failure to include requested items will delay the evaluation of your revision.

We require:
1) A .docx formatted version of the manuscript text (including legends for main figures, EV figures and tables). Please make sure that the changes are highlighted to be clearly visible. Alternatively you may choose to submit your manuscript as a LaTeX file.

2) Individual production quality figure files as .eps, .tif, .jpg (one file per figure). For guidance, download the 'Figure Guide PDF' (https://media.springernature.com/original/springer-cms/rest/v1/content/27825798/data/v1).

3) At EMBO Press we ask authors to provide source data for the main figures. Our source data coordinator will contact you to discuss which figure panels we would need source data for and will also provide you with helpful tips on how to upload and organize the files.

4) A .docx formatted letter INCLUDING the reviewers' reports and your detailed point-by-point responses to their comments. As part of the EMBO Press transparent editorial process, the point-by-point response is part of the Peer Review File (PRF), which will be published alongside your paper.

5) A complete author checklist, which you can download from our author guidelines (https://link.springer.com/journal/44320/submission-guidelines#cms). Please insert information in the checklist that is also reflected in the manuscript. The completed author checklist will also be part of the PRF.

6) Please note that all corresponding authors are required to supply an ORCID ID for their name upon submission of a revised manuscript.

7) It is mandatory to include a 'Data Availability' section after the Materials and Methods. Before submitting your revision, primary datasets produced in this study need to be deposited in an appropriate public database, and the accession numbers and database listed under 'Data Availability'. Please remember to provide a reviewer password if the datasets are not yet public (see https://link.springer.com/partners/embo-press/editorial-policies#Data%20availability%20statement).

In case you have no data that requires deposition in a public database, please state so in this section as follows: "This study includes no data deposited in external repositories". Note that the Data Availability Section is restricted to new primary data that are part of this study.

8) All Materials and Methods need to be described in the main text using our 'Structured Methods' format, which is required for all research articles. According to this format, the Methods section includes a Reagents and Tools Table (listing key reagents, experimental models, software and relevant equipment and including their sources and relevant identifiers) followed by a Methods and Protocols section describing the methods using a step-by-step protocol format. The aim is to facilitate adoption of the methodologies across labs. Please upload the Reagents and Tools table as a separate document when submitting your revised manuscript. More information on how to adhere to this format as well as a downloadable template (.docx) for the Reagents and Tools Table can be found in our author guidelines: https://link.springer.com/journal/44320/submission-guidelines#cms

9) For data quantification: please specify the name of the statistical test used to generate error bars and p-values, the number (n) of independent experiments (specify technical or biological replicates) underlying each data point and the test used to calculate p-values in each figure legend. The figure legends should contain a basic description of n, p-values and the test applied. Graphs must include a description of the bars and the error bars (s.d., s.e.m.). Please provide exact p-values (in either the figure or figure legend).

10) Our journal encourages inclusion of *data citations in the reference list* to directly cite datasets that were re-used and obtained from public databases. Data citations in the article text are distinct from normal bibliographical citations and should directly link to the database records from which the data can be accessed. In the main text, data citations are formatted as follows: "Data ref: Smith et al, 2001" or "Data ref: NCBI Sequence Read Archive PRJNA342805, 2017". In the Reference list, data citations must be labeled with "[DATASET]". A data reference must provide the database name, accession number/identifiers and a resolvable link to the landing page from which the data can be accessed at the end of the reference. Further instructions are available at .

11) We replaced Supplementary Information with Expanded View (EV) Figures and Tables that are collapsible/expandable online. EV Figures should be cited as 'Figure EV1, Figure EV2" etc... in the text and their respective legends should be included in the main text after the legends of regular figures.
- For the figures that you do NOT wish to display as Expanded View figures, they should be bundled together with their legends in a single PDF file called *Appendix*, which should start with a short Table of Content. Appendix figures should be referred to in the main text as: "Appendix Figure S1, Appendix Figure S2" etc.
- Additional Tables/Datasets should be labeled and referred to as Table EV1, Dataset EV1, etc. Legends should be provided in a separate tab in case of .xls files. Alternatively, the legend can be supplied as a separate text file (README) and zipped together with the Table/Dataset file.
See detailed instructions here:
https://link.springer.com/journal/44320/submission-guidelines#cms

12) Author contributions: CRediT has replaced the traditional author contributions section because it offers a systematic machine-readable author contributions format that allows for more effective research assessment. Please remove the Authors Contributions from the manuscript and use the free text boxes beneath each contributing author's name in our system to add specific details on the author's contribution. More information is available in our guide to authors.

13) Disclosure statement and competing interests: We updated our journal's competing interests policy in January 2022 and request authors to consider both actual and perceived competing interests. Please review the policy https://link.springer.com/partners/embo-press/editorial-policies#Competing%20interest%20disclosures and update your competing interests if necessary.

14) Every published paper now includes a 'Synopsis' to further enhance discoverability. Synopses are displayed on the journal webpage and are freely accessible to all readers. They include a short stand first (maximum of 300 characters, including space) as well as 2-5 one-sentences bullet points that summarizes the paper. Please write the bullet points to summarize the key NEW findings. They should be designed to be complementary to the abstract - i.e. not repeat the same text. We encourage inclusion of key acronyms and quantitative information (maximum of 30 words / bullet point). Please use the passive voice. Please attach these in a separate file or send them by email, we will incorporate them accordingly.

Please also suggest a striking image or visual abstract to illustrate your article as a PNG file 550 px wide x 300 px high.

Please note that these would be the final versions and changes during proofing are usually not allowed.

15) As part of the EMBO Publications transparent editorial process initiative (see our policy here: https://link.springer.com/partners/embo-press/editorial-policies#Peer%20review), Molecular Systems Biology will publish online a Peer Review File (PRF) to accompany accepted manuscripts.
In the event of acceptance, this file will be published in conjunction with your paper and will include the anonymous referee reports, your point-by-point response and all pertinent correspondence relating to the manuscript. Let us know whether you agree with the publication of the PRF and as here, if you want to remove or not any figures from it prior to publication.
Please note that the Author checklist will be published at the end of the PRF.

Molecular Systems Biology has a "scooping protection" policy, whereby similar findings that are published by others during review or revision are not a criterion for rejection. Should you decide to submit a revised version, I do ask that you get in touch after three months if you have not completed it, to update us on the status.

Yours sincerely,

Poonam Bheda, PhD
Scientific Editor
Molecular Systems Biology

***

Rev_Com_number: RC-2026-03377
New_manu_number: MSB-2026-13739-T
Corr_author: Parker
Title: Quantitative interactome mapping of skeletal muscle insulin resistance

Reviewer #1 (Evidence, reproducibility and clarity (Required)):

Summary

Ng et al. use a combination of quantitative (structural) proteomics tools to study the effect of insulin resistance (IR) on protein interactions in mouse muscle cells (C2C12 myotubes) and muscle tissue. First, the authors use protein correlation profiling (PCP; co-fractionation) to separate protein complexes from C2C12 cells with and without induced insulin resistance. The PCP data is then used to reconstruct protein complexes and networks based on binary interactions and to uncover changes upon IR, this is illustrated by several examples (Fig. 1). As an orthogonal method quantitative cross-linking (XL) is used to study protein interactions in C2C12 cells. A multidimensional enrichment/fractionation scheme is designed to increase the number of cross-links/protein-protein interactions (PPIs), and again interaction networks are generated and changes between no IR vs. IR conditions assessed (Fig. 2). The XL approach is then carried forth into a mouse model of IR. Muscle tissue of mice fed on regular chow or on a high-fat diet are compared (Fig. 3). Selected XL data is validated on known structures and quantitative data from PCP and XL is compared to integrate the different interactome data (Fig. 4). Finally, the interactions of PDIA6, a key protein found to be affected by IR, are studied in more detail in the mouse model. The effect of overexpression of PDIA6 is studied using different readouts, including redox proteomics (Fig. 5). These data connect the redox imbalance / cysteine oxidation with the role of PDIA6 in insulin resistance.

Overall, the manuscript is impressive with respect to the methodological effort undertaken, generating and combining many large-scale proteomics data sets, both from a cell line and from mouse tissue. The large amount of data accumulated can be seen as a strength and as a weakness, because it is impossible to follow up on all findings (changes of interactions induced by IR observed in any sample and with any method). Nevertheless, the PDIA6 example was evaluated in more detail and with dedicated follow-up experiments. The conclusions from this experiment are plausible and presented logically. It is, however, difficult for a reviewer to quickly judge whether there would have been more promising leads for validation experiments than PDIA6.

Given the large amount of data already generated, I do not have suggestions for additional experiments. Instead, some analysis and interpretation of existing data need further clarification.

Major comments

The search strategy and statistical treatment of the cross-linking data need to be explained more clearly. The authors write that the data were searched with pLink2 and the FDR was controlled at 1%. However, it remains unclear whether the FDR was controlled at the level of peptide/cross-link-spectral-matches (PSMs/CSMs), or at the level of non-redundant site pairs or even protein-protein interactions for inter-protein links, which would be more appropriate. Controlling the FDR only at the PSM/CSM level will lead to an inflation of false positives when aggregating results at the interaction level. Moreover, it remains unclear whether all data sets were searched together or whether individual data sets or subsets of the entire data were searched individually. For example, if single files or only files belonging to a certain higher-order fraction would be searched individually, this would again lead to an underestimation of false positives. What is particularly noteworthy in this context is that in a typical dataset inter-protein links are underrepresented compared to intra-protein links for statistical reasons, while the numbers in this manuscript are much more balanced (almost equal numbers in the cell line data set, and even more inter-protein links than intra-protein links in the tissue). The authors should consider articles that discuss FDR control in XL-MS, for example by the Rappsilber group. Minimally, more details about what was searched together and at what level the FDR was controlled need to be provided.

This is a good suggestion and we have reprocessed our data with xiFDR from the Rappsilber group as suggested. We have included various additional FDR based on peptide pairs, residue pairs and protein pairs. Cross-link converter (CroCO), developed by Julian Bender and Carla Schmidt, was used to combine all pLink2 reports into a file containing peptide sequence, peptide position, cross link site and pLink2 score. This was then used as an input into xiFDR where FDR was applied at the peptide pair (<0.05), residue pair (<0.1) and protein interaction (<0.05) level. We now report the protein interaction that pass this FDR and the associated xiFDR score at each level in Dataset EV4 (C2C12) and Dataset EV6 (EDL skeletal muscle). Note, the xiFDR analysis was done with the qXL-MS analysis of both C2C12 and mouse skeletal muscle separately. As suggested by the reviewer, this led to a decrease in the percentage of intra- vs inter-crosslinked peptides. For example, at only 1% CSM FDR there were 4,944/11,295 inter-protein XL peptides (44%) while after applying xiFDR filtering this dropped to 2,345/8,671 inter-protein XL peptides (27%). We have updated Fig 2C (C2C12) and Fig 3F (EDL skeletal muscle) to show the results from xiFDR filtering.

While the added xiFDR analysis reduced the number of significantly regulated XL-peptides following statistical analysis in both C2C12 and EDL qXL-MS datasets, a pathway analysis revealed no major differences in enrichment. Importantly, PDIA6 was still identified as a major hub containing dysregulated interactions. Therefore, we kept all the original

quantitative analysis of XL peptides at a CSM <1% FDR. The added xiFDR analysis has been added to Dataset EV4/EV6 and hence readers can further filter the quantitative results.

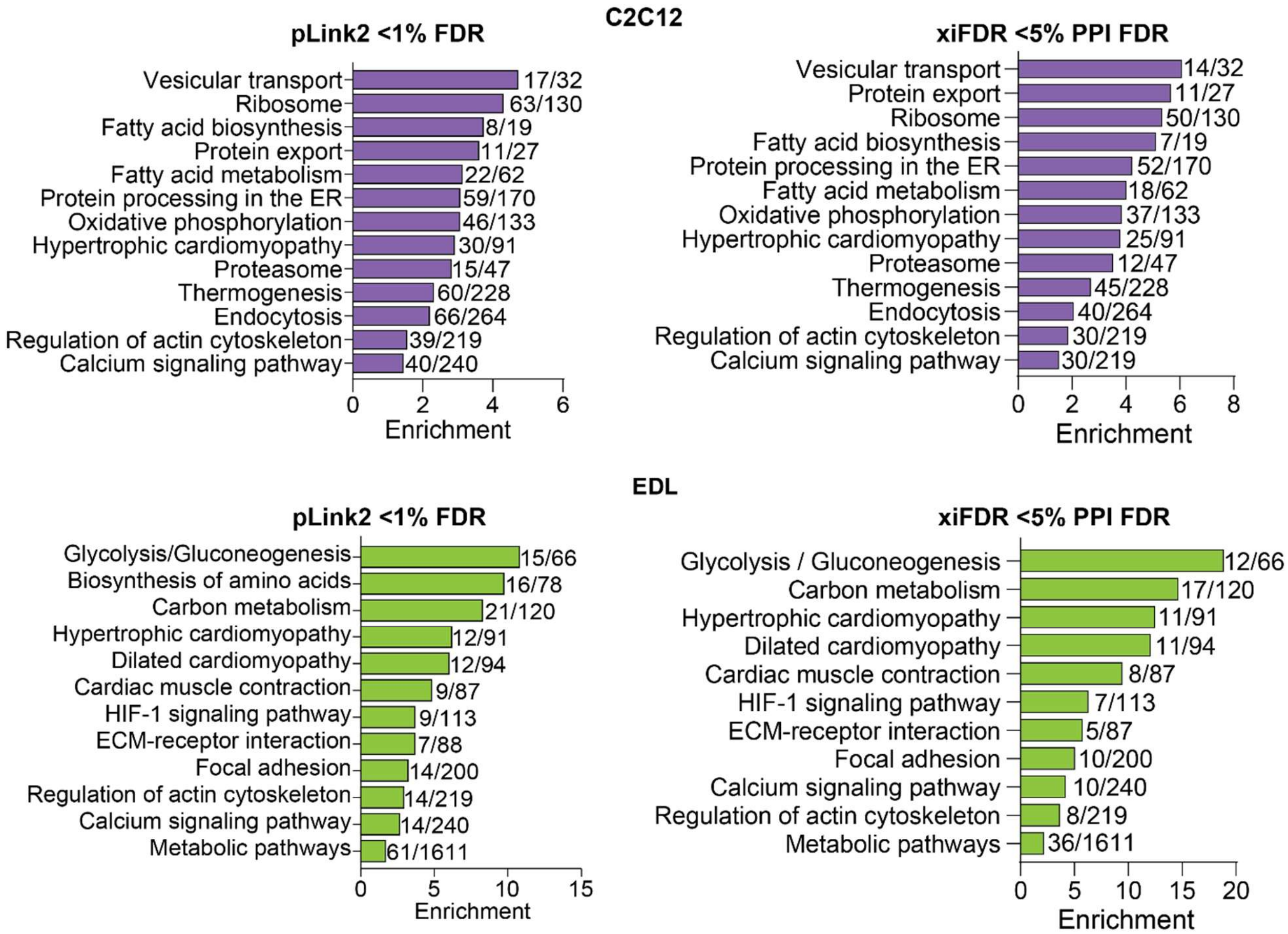

We have provided additional information on how the data sets were searched in batches. This was required because processing 132 files from the C2C12 qXL-MS and 192 files from the skeletal muscle qXL-MS studies each took >1 month to process on our severer with 128 cores and 1TB of RAM despite restricting database size to ~12,000 entries. We have updated the methods on line 1009:

"Due to the large search space and computational requirements, data were processed in 12 x fractions of C18BEH batches for each SEC fraction where each batch took ~3 days to search on an AMD EPYC 9474F 128-Core 3.6 GHz Processor with 1TB RAM. The C2C12 qXL-MS data included 11 SEC fractions x 12 C18BEH fractions = 132 .raw files while the skeletal muscle qXL-MS data included 16 SEC fractions x 12 C18BEH fractions = 192 .raw files. The resulting PSMs for

C2C12 or skeletal muscle data were combined and post-processed (separately) with xiFDR."

On a similar note, it has been discussed in the literature that validating large-scale XL data on selected structures of complexes is a poor proxy for accurate FDR control, as such complexes are commonly not representative for more transient or substoichiometric PPIs, or PPIs involving low-abundant proteins.

This is a good point and we have removed any mention of the mapping of XL peptide onto resolved structures as validation.

Minor comments

Figure 1: CCT is a complex composed of eight subunits, but only seven are shown. What happened to the remaining one (CCT6)?

We identified both CCT6A and CCT6B but the identifications did not pass our filtering that required identification in at least 2/3 biological replicates.

The authors performed a redox proteomics experiment in a PDIA6 overexpression system. However, the statement in the Conclusion section that PDIA6 overexpression promotes disulfide bond formation in interacting proteins is not directly justified because the method only quantifies cysteine oxidation, not S-S bond formation directly.

This is a good point and we have modified the conclusion section on line 678:

"…the ER chaperone PDIA6 where its overexpression negatively impacted insulin stimulated glucose uptake while promoting cysteine oxidation in key regions of its interacting proteins."

All supplementary data is not provided in an independent repository, but in a repository of the authors' institution. It is unclear whether the data could be accessed anonymously. Proteomics data need to be provided in an independent, community accepted repository such as from the members of the proteomeXchange consortium (PRIDE etc.).

We have uploaded all data to the PRIDE ProteomeXchange consortium. Accession and reviewer access is provided in the Data Availability section, and will be publicly released upon

acceptance of the manuscript prior to publication.

A clear description of what is shown in the SI tables is missing, e.g. in the form of figure legends. In their present form, SI data are difficult to interpret. For example, I did not find information about cross-link identifications, only quantitative data on cross-link changes. However, if the identification of a cross-link is not confident in the first place (see my comments above), then the quantification will be irrelevant.

We have provided more comprehensive legends for the Expanded View Datasets.

Reviewer #1 (Significance (Required)):

The study is one of only a few so far that combines PCP and XL on such a large scale for a mammalian system. There are also very few studies of cross-linking on tissue. Therefore, from a methodological point of view, the study is highly innovative. The application to the muscle cell system and insulin resistance as a biological research question is furthermore very novel. As such, the study is valuable to different communities - those developing and refining experimental methods and those using them to uncover regulatory mechanisms. Another strength is that the authors made serious efforts at each step to optimize the XL method and adapt it to their sample types of interest.

The wealth of data is both a strength and a weakness of the work. Inevitably, a reviewer might argue that some aspects of the work could have been done differently. Unless someone spends a lot of time going deep into the result tables, it will be difficult to make constructive suggestions on additional targets for further investigation. Nevertheless, some statistical aspects of data analysis need to be clarified, and parts of the data analysis might need to be repeated. This, in turn, may require some reinterpretation of findings related to the XL data.

Advances: Conceptual, methodological, mechanistic

Audience: Specialized, basic research, translational

Reviewer expertise

My background is in proteomics, structural proteomics, mass spectrometry, analytical sciences, experimental methodology, and computational data analysis. I have general knowledge of biological processes, but I am not an expert on insulin resistance.

Reviewer #2 (Evidence, reproducibility and clarity (Required)):

Quantitative interactome mapping of skeletal muscle insulin resistance
Ng et al present a series of proteomics/interactomics studies in skeletal muscle to identity insulin regulated complexes/interactions and changes ot these in insulin resistant muscle. More mechanistically, the Authors focus on changes in interactions involving chaperones in the ER/SR, presenting interesting data on the effect of PDIA6 overexpression alters insulin sensitivity in muscle ex vivo.

Major Comments:
The section entitled "Validating the regulation of PPIs with insulin resistance in C2C12 myotubes with quantitative XL-MS". This is not really a validation of th previous data as presented, but more an orthologous assay that helped pinpoint the interest in the ER. Suggest adjusting the title.

We have changed the title of this section to:

"Probing the regulation of direct PPIs with insulin resistance in C2C12 myotubes with quantitative XL-MS"

We have also removed any mention of 'validation' throughout the results including the mapping of XL peptides onto tertiary structures as suggested by Reviewer 1.

Figure 3B - the "decrease" in AS160 pS588 regulation appears to be due to increased basal, not decreased phosphorylation in after insulin. This should be commented on or clarified.

This is a good point and we have changed the description of the results on line 340 to indicate a change in the fold-change of phosphorylation:

"…and reduced <u>fold-change</u> of phosphorylation on pS473 on Akt and pS588 on AS160 (Fig. 3A-C)"

PDIA6 is down-regulated in muscle from people with T2D - so why did the authors decide to overexpress PDIA6? I note this rationale is explained in the discussion, and could be articulated better in the results.

We have modified the results on line 512 to provide greater rationale for the choice of an over-expression model.

“We selected an over-expression model with the hypothesis that increased PDIA6 would improve proteostasis and have positive effects on insulin sensitivity.”

Figure 5J and K. The TA muscles are substantially larger from PDIA6 OE mice. Are the muscle fibres also larger? Tbhis relates to the normalisation of data in K. This appears to be normalised to g tissue. If so, is the difference between control, and OE mice being driven by the increase in muscle mass - with uptake per muscle or per fibre the same?

This is a good point. Unfortunately, we did not fix the muscles to allow assessment of muscle fiber size by e.g histology analysis. Hence, we are unable to assess this. The data in Fig. 5K was normalized to the proportion of protein content in the sample used for scintillation counting, not the muscle weight. This is the standardized approach used in the following protocol and several labs around the world (https://www.jove.com/t/61398/measurement-insulin-contraction-stimulated-glucose-uptake-isolated)

We have modified the y-axis title to read “µmol/g protein/hr”

Minor Comments:
For the PCP-MS data form C2C12 cells. The authors use an analysis of AUC to assess protein abundance, which, as they state, is important for chronic treatments if total protein is not separately quantified. However, the analysis of changes in protein distribution is less clear from the text in the results section. Intuitively, a profile that is normalised to total intensity in all fractions would provide a protein abundance-independent read-out for changes in protein distribution. Does the "local analysis" capture this same information? Could the Authors provide a little more information here?

Yes, the local analysis essentially captures changes in the distribution of proteins migrating through blue native-PAGE independently of changes in protein abundance. We have included a further description on line 124, and also updated the legend of Dataset EV3

Figure 1M - are the Authors sure that VPS41 should be in this panel. It doesn't seem to be insulin regulated, and the arrow appears to refer to movement between insulin sensitive and insulin resistant.

We thank the reviewer for pointing this out. VPS41 had both a subtle but significant shift with insulin stimulation in the control cells, and also a more prominent shift with the induction of insulin resistance. We have annotated this in Figure 1M with additional asterisks.

Figure 1N - "This includes an array of TBC1 domain-containing proteins (TBC1D15, 195 TBC1D17, TBC1D8B) that are consistently reduced with IR". Do the Authors mean the abundance was less, or that complex formation was reduced?

There were both reduced abundances with IR and local changes in migration profiles with insulin stimulation. We have modified the results on line 192:

"This includes an array of TBC1 domain-containing proteins (TBC1D15, TBC1D17, TBC1D8B) that displayed both reduced abundance with IR and changes in the migration profiles including shifts with acute insulin stimulation."

Optional. In general, there is a lot of text discussing the literature around proteins highlighted in the analysis. This is useful to an extent, but the Authors might consider streamlining this a little (perhaps moving some of the information ot supp tables?).

We have summarized some of these discussions.

On line 203, we have reduced the discussion of the link between TBC1D8B to RAB11.

Why do the Authors think the crosslinking MS was not able to capture acute PPI changes like the PCP-MS was?

We speculate this may be due to both the low stoichiometry of cross-linking and low stoichiometry of PPIs that are modulated with acute insulin stimulation. For example, one hypothesis is rapid changes in phosphorylation drive changes in PPI. Phosphorylation generally occurs at reasonably low stoichiometric levels. E.g <10% of Akt is phosphorylated following acute stimulation with insulin (https://pubmed.ncbi.nlm.nih.gov/22207758/). Hence, only a small population of the protein pool changes PPI which is consistent with overall subtle changes in the migration profiles observed with PCP-MS following acute insulin stimulation. If we assume that cross-linking also occurs at low stoichiometric levels, the chances of cross-linking and identifying this small pool of PPIs modulated with acute insulin stimulation is low.

For the EDL crosslinking data. Are the Authors able to provide a comparison with C2C12 data -

to highlight the differences and similarities between tissue and the cell model? This may be a challenge if the authors think most differences may be technical.

We have integrated the C2C12 and EDL qXL-MS data with the C2C12 PCP-MS data and provide these results in Dataset EV8. We also include a list of exact XL-peptides identified in the C2C12 and EDL qXL-MS data in Dataset EV6.

Please check - "reduces free-glycerol levels essential for fatty acid synthesis". Glycerol does not directly contribute to FA synthesis. But is needed for triglyceride synthesis.

We have modified this sentence on line 462:

"…and indirectly reduces free-glycerol levels essential for <u>triglyceride</u> synthesis"

Do the Authors think that the change in PDIA6 interactions may be a general/indirect indication of changes in ER redox and/or protein misfolding in insulin resistance?

This is an interesting question and given we identified ~18 proteins with direct interactions with PDIA6 that were dysregulated with IR including BiP, a master regulator of ER stress, we conclude that there is a likely a general change in ER redox/protein folding in IR.

Is PDIA6 an ER luminal protein? If so, it being phosphorylated is interesting.

Yes, the Human Protein Atlas suggests that PDIA6 is mainly located to the ER and we agree that phosphorylation of this protein is interesting particularly given the complex changes with exercise/insulin stimulation (Fig 5B-C).

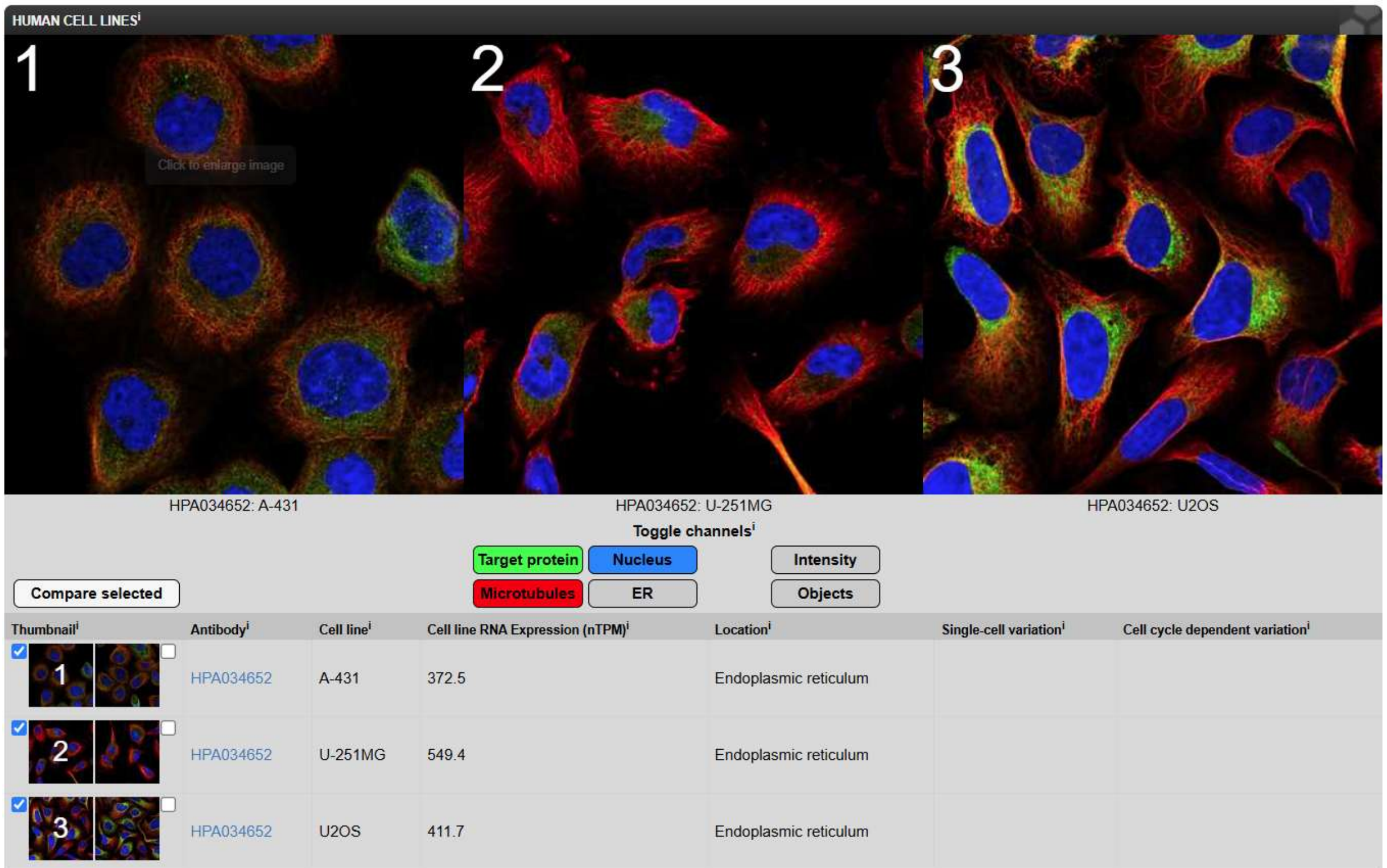

Referees cross-commenting

Similarly, reviewer #1 raises important points on the description of key parts of the analysis, that will need to be addressed. I think we agree that the manuscript encompasses a great deal of data, and that it is somewhat difficult to follow why PDIA6 was selected for validation. Overall, the reviews pick up on different aspects of the manuscript that could be improved.

28th May 2026

Manuscript Number: MSB-2026-13739R
Title: Quantitative interactome mapping of skeletal muscle insulin resistance

Dear Dr. Parker,

Thank you for the submission of your revised manuscript to Molecular Systems Biology. We have now received the enclosed reports from the referees that were asked to re-assess it. As you will see the reviewers are now globally supportive and I am pleased to inform you that we will be able to accept your manuscript pending the following final amendments:

1) In the main manuscript file, please include keywords to max. 5.
2) Please format the Data availability section according to the example below and ensure that all proteomics datasets have now been publicly released.
"The datasets and computer code produced in this study are available in the following databases:
- Chip-Seq data: Gene Expression Omnibus GSE46748 (https://www.ncbi.nlm.nih.gov/geo/query/acc.cgi?acc=GSE46748)
- Modeling computer scripts: GitHub (https://github.com/SysBioChalmers/GECKO/releases/tag/v1.0)
- [data type]: [full name of the resource] [accession number/identifier] ([doi or URL or identifiers.org/DATABASE:ACCESSION])"
3) Please include a "Disclosure and competing interests statement" after the Acknowledgements. Employment in a biotech company should be included in this statement. We updated our journal's competing interests policy in January 2022 and request authors to consider both actual and perceived competing interests. Please review the policy https://link.springer.com/partners/embo-press/editorial-policies#Competing%20interest%20disclosures and update your competing interests if necessary.
4) Please remove the author contributions from the manuscript and specify author contributions in our submission system. CRediT has replaced the traditional author contributions section because it offers a systematic machine-readable author contributions format that allows for more effective research assessment. You are encouraged to use the free text boxes beneath each contributing author's name to add specific details on the author's contribution. More information is available in our guide to authors: https://link.springer.com/journal/44320/submission-guidelines#cms-Manuscript-organisation-and-formatting
5) Please correct the reference citation in the reference list such that where there are more than 10 authors on a paper, only the first 10 should be listed, followed by "et al.". DOIs should only be used for preprints and datasets that have not been published yet. Please check "Author Guidelines" for more information: https://link.springer.com/journal/44320/submission-guidelines#cms-Reference-guidelines
6) Our journal encourages inclusion of *data citations in the reference list* to directly cite datasets that were re-used and obtained from public databases. Data citations in the article text are distinct from normal bibliographical citations and should directly link to the database records from which the data can be accessed. In the main text, data citations are formatted as follows: "Data ref: Smith et al, 2001" or "Data ref: NCBI Sequence Read Archive PRJNA342805, 2017". In the Reference list, data citations must be labeled with "[DATASET]". A data reference must provide the database name, accession number/identifiers and a resolvable link to the landing page from which the data can be accessed at the end of the reference. Further instructions are available at: https://link.springer.com/journal/44320/submission-guidelines#cms-Reference-guidelines
7) In the Methods, please take care of the following:
- Cell lines: Please be sure to include a sentence in the Methods as to whether or not the C2C12 cell line was recently authenticated and tested for mycoplasma contamination. Please also be sure to update the Author Checklist with this information and where it can be found in the manuscript.
- Please ensure that a statement on whether or not blinding was done is included in the Methods even if no blinding was done. Please also be sure to update the Author Checklist with this information and where it can be found in the manuscript.
8) Please place individual sections of the manuscript in the following order: Title page - Abstract & Keywords - Introduction - Results - Discussion - Methods - Data Availability - Acknowledgements - Disclosure and Competing Interests Statement - References - Figure Legends - (Main Tables with legends if applicable) - Expanded View Figure Legends.
9) For the figures and figure legends, please take care of the following:
- Please remove all figures from main manuscript file and leave only main figure legends placed after the references.
- Please note that the exact p values are not provided in the legends of 1C,F,H,I,K,M,N; 3A,C; 5D,H,I,J,K,M,N,P.
- Please note that information related to n is missing in the legends of 1C,K,L; 3A,C; 5H,I,J,K,M,N.
10) Please upload all Tables EV1-EV9 as one .xsl file per table and rename them to Dataset EV1-EV9. The source file names, titles, legends and manuscript callouts all need to be updated to Dataset EV1-EV9 instead of Expanded View Tables, legends should be removed from the manuscript and included in the corresponding file in a separate tab/sheet in each Excel file. Datasets should be uploaded as individual Excel files, not zipped together. There is also a callout for a 'Supplemental Table S8' that should be updated to Dataset EV8.
11) Synopsis image: Please provide a graphic that summarises the main findings of the manuscript on a glance and upload it as a high-resolution jpeg file 550 pixels wide x 300 pixels high.
12) As part of the EMBO Publications transparent editorial process initiative (see our policy here: https://www.embopress.org/transparent-process#Review_Process), Molecular Systems Biology will publish online a Peer

Review File (PRF) to accompany accepted manuscripts. This file will be published in conjunction with your paper and will include the anonymous referee reports, your point-by-point response and all pertinent correspondence relating to the manuscript. Let us know whether you agree with the publication of the PRF and as here, if you want to remove or not any figures from it prior to publication. Please note that the Authors checklist will be published at the end of the PRF.
13) After your paper is published, we may promote it on social media. If you have any handles or hashtags for Bluesky you would like included, please let us know.
14) Please provide a point-by-point letter INCLUDING my comments and your detailed responses (as Word file).

I look forward to reading a new revised version of your manuscript as soon as possible.

Yours sincerely,

Poonam Bheda, PhD
Scientific Editor
Molecular Systems Biology

Reviewer #1:

In this revised version based on a reviewed preprint, the authors have made substantial changes in response to my comments.

Specifically, the analysis of cross-linking data was enhanced by improved false discovery rate control, which is more in line with community recommendations while still preserving the main biological conclusions. In addition, more experimental details have been provided in the revised manuscript, and the data has been deposited in a public repository. The layout of SI tables has been improved and other minor changes to the text have been implemented.

Reviewer #2:

The Authors have responded to and/or addressed my comments. I have no further comments.

1) In the main manuscript file, please include keywords to max. 5.

We have included 5 keywords.

2) Please format the Data availability section according to the example below and ensure that all proteomics datasets have now been publicly released.
"The datasets and computer code produced in this study are available in the following databases:
- Chip-Seq data: Gene Expression Omnibus GSE46748 (https://www.ncbi.nlm.nih.gov/geo/query/acc.cgi?acc=GSE46748)
- Modeling computer scripts: GitHub (https://github.com/SysBioChalmers/GECKO/releases/tag/v1.0)
- [data type]: [full name of the resource] [accession number/identifier] ([doi or URL or identifiers.org/DATABASE:ACCESSION])"

We have updated the data availability section.

3) Please include a "Disclosure and competing interests statement" after the Acknowledgements. Employment in a biotech company should be included in this statement. We updated our journal's competing interests policy in January 2022 and request authors to consider both actual and perceived competing interests. Please review the policy https://link.springer.com/partners/embo-press/editorial-policies#Competing%20interest%20disclosures and update your competing interests if necessary.

We have included a Disclosure and competing interests statement

4) Please remove the author contributions from the manuscript and specify author contributions in our submission system. CRediT has replaced the traditional author contributions section because it offers a systematic machine-readable author contributions format that allows for more effective research assessment. You are encouraged to use the free text boxes beneath each contributing author's name to add specific details on the author's contribution. More information is available in our guide to authors: https://link.springer.com/journal/44320/submission-guidelines#cms-Manuscript-organisation-and-formatting

We have included author contributions in the submission system and removed from manuscript.

5) Please correct the reference citation in the reference list such that where there are more than 10 authors on a paper, only the first 10 should be listed, followed by "et al.". DOIs should only be used for preprints and datasets that have not been published yet. Please check "Author Guidelines" for more information: https://link.springer.com/journal/44320/submission-guidelines#cms-Reference-guidelines

We have updated the references.

6) Our journal encourages inclusion of *data citations in the reference list* to directly cite datasets that were re-used and obtained from public databases. Data citations in the article text are distinct

from normal bibliographical citations and should directly link to the database records from which the data can be accessed. In the main text, data citations are formatted as follows: "Data ref: Smith et al, 2001" or "Data ref: NCBI Sequence Read Archive PRJNA342805, 2017". In the Reference list, data citations must be labeled with "[DATASET]". A data reference must provide the database name, accession number/identifiers and a resolvable link to the landing page from which the data can be accessed at the end of the reference. Further instructions are available at: https://link.springer.com/journal/44320/submission-guidelines#cms-Reference-guidelines

We have not re-used any datasets.

7) In the Methods, please take care of the following:
- Cell lines: Please be sure to include a sentence in the Methods as to whether or not the C2C12 cell line was recently authenticated and tested for mycoplasma contamination. Please also be sure to update the Author Checklist with this information and where it can be found in the manuscript.

We have updated the methods and the author checklist confirming that the cells were free of mycoplasma.

- Please ensure that a statement on whether or not blinding was done is included in the Methods even if no blinding was done. Please also be sure to update the Author Checklist with this information and where it can be found in the manuscript.

We have included a statement that no blinding was performed in the Methods and we have updated the author checklist.

8) Please place individual sections of the manuscript in the following order: Title page - Abstract & Keywords - Introduction - Results - Discussion - Methods - Data Availability - Acknowledgements - Disclosure and Competing Interests Statement - References - Figure Legends - (Main Tables with legends if applicable) - Expanded View Figure Legends.

We have reordered the manuscript.

9) For the figures and figure legends, please take care of the following:
- Please remove all figures from main manuscript file and leave only main figure legends placed after the references.

We have removed figure and placed legends after the references.

- Please note that the exact p values are not provided in the legends of 1C,F,H,I,K,M,N; 3A,C; 5D,H,I,J,K,M,N,P.
- Please note that information related to n is missing in the legends of 1C,K,L; 3A,C; 5H,I,J,K,M,N.

We have included n values and exact p-values in figure legends

10) Please upload all Tables EV1-EV9 as one .xsl file per table and rename them to Dataset EV1-EV9. The source file names, titles, legends and manuscript callouts all need to be updated to Dataset EV1-EV9 instead of Expanded View Tables, legends should be removed from the manuscript and included

in the corresponding file in a separate tab/sheet in each Excel file. Datasets should be uploaded as individual Excel files, not zipped together. There is also a callout for a 'Supplemental Table S8' that should be updated to Dataset EV8.

All edits have been made.

11) Synopsis image: Please provide a graphic that summarises the main findings of the manuscript on a glance and upload it as a high-resolution jpeg file 550 pixels wide x 300 pixels high.

We uploaded a Synopsis image

12) As part of the EMBO Publications transparent editorial process initiative (see our policy here: https://www.embopress.org/transparent-process#Review_Process), Molecular Systems Biology will publish online a Peer Review File (PRF) to accompany accepted manuscripts. This file will be published in conjunction with your paper and will include the anonymous referee reports, your point-by-point response and all pertinent correspondence relating to the manuscript. Let us know whether you agree with the publication of the PRF and as here, if you want to remove or not any figures from it prior to publication. Please note that the Authors checklist will be published at the end of the PRF.

We agree to publish PRF as is.

13) After your paper is published, we may promote it on social media. If you have any handles or hashtags for Bluesky you would like included, please let us know.

@benparkerlab.bsky.social

14) Please provide a point-by-point letter INCLUDING my comments and your detailed responses (as Word file).

As attached

9th Jun 2026
Manuscript number: MSB-2026-13739RR
Title: Quantitative interactome mapping of skeletal muscle insulin resistance

Dear Dr. Parker,

Thank you again for sending us your revised manuscript. We are now satisfied with the modifications made and I am pleased to inform you that your paper has been accepted for publication.

Your manuscript will be processed for publication by EMBO Press. It will be copy edited and you will receive page proofs prior to publication. Please note that you will be contacted by Springer Nature Author Services to complete licensing and payment information.

You may qualify for financial assistance for your publication charges - either via a Springer Nature fully open access agreement or an EMBO initiative. Check your eligibility: https://link.springer.com/journal/44320/how-to-publish-with-us

Should you be planning a Press Release on your article, please notify the production team via this form in order to coordinate publication and release dates: https://forms.cloud.microsoft/e/eH4A8JVP6g

If you have any questions, please do not hesitate to contact the Editorial Office. Thank you for your contribution to Molecular Systems Biology.

Yours sincerely,

Sincerely,

Poonam Bheda, PhD
Scientific Editor
Molecular Systems Biology

>>> Please note that it is Molecular Systems Biology policy for the transcript of the editorial process (containing referee reports and your response letter) to be published as an online supplement to each paper. If you do NOT want this, you will need to inform the Editorial Office via email immediately. More information is available here: https://link.springer.com/partners/embo-press/editorial-policies#Peer%20review

EMBO Press Author Checklist

Corresponding Author Name: Benjamin L. Parer
Journal Submitted to: Molecular Systems Biology
Manuscript Number: MSB-2026-13739R

USEFUL LINKS FOR COMPLETING THIS FORM
The EMBO Journal - Author Guidelines
EMBO Reports - Author Guidelines
Molecular Systems Biology - Author Guidelines
EMBO Molecular Medicine - Author Guidelines

Reporting Checklist for Life Science Articles (updated January
This checklist is adapted from Materials Design Analysis Reporting (MDAR) Checklist for Authors. MDAR establishes a minimum set of requirements in transparent reporting in the life sciences (see Statement of Task: 10.31222/osf.io/9sm4x). Please follow the journal's guidelines in preparing your
Please note that a copy of this checklist will be published alongside your article.

Abridged guidelines for figures
1. Data
The data shown in figures should satisfy the following conditions:
- the data were obtained and processed according to the field's best practice and are presented to reflect the results of the experiments in an accurate and unbiased manner.
- ideally, figure panels should include only measurements that are directly comparable to each other and obtained with the same assay.
- plots include clearly labeled error bars for independent experiments and sample sizes. Unless justified, error bars should not be shown for technical
- if n<5, the individual data points from each experiment should be plotted. Any statistical test employed should be justified.
- Source Data should be included to report the data underlying figures according to the guidelines set out in the authorship guidelines on Data

2. Captions
Each figure caption should contain the following information, for each panel where they are relevant:
- a specification of the experimental system investigated (eg cell line, species name).
- the assay(s) and method(s) used to carry out the reported observations and measurements.
- an explicit mention of the biological and chemical entity(ies) that are being measured.
- an explicit mention of the biological and chemical entity(ies) that are altered/varied/perturbed in a controlled manner.
- the exact sample size (n) for each experimental group/condition, given as a number, not a range;
- a description of the sample collection allowing the reader to understand whether the samples represent technical or biological replicates (including how many animals, litters, cultures, etc.).
- a statement of how many times the experiment shown was independently replicated in the laboratory.
- definitions of statistical methods and measures:
  - common tests, such as t-test (please specify whether paired vs. unpaired), simple χ2 tests, Wilcoxon and Mann-Whitney tests, can be unambiguously identified by name only, but more complex techniques should be described in the methods section;
  - are tests one-sided or two-sided?
  - are there adjustments for multiple comparisons?
  - exact statistical test results, e.g., P values = x but not P values < x;
  - definition of 'center values' as median or average;
  - definition of error bars as s.d. or s.e.m.

Please complete ALL of the questions below.
Select "Not Applicable" only when the requested information is not relevant for your study.

Materials

Newly Created Materials	Information included in the manuscript?	In which section is the information available? (Reagents and Tools Table, Materials and Methods, Figures, Data Availability Section)
New materials and reagents need to be available; do any restrictions apply?	Not Applicable	

Antibodies	Information included in the manuscript?	In which section is the information available? (Reagents and Tools Table, Materials and Methods, Figures, Data Availability Section)
For **antibodies** provide the following information:<br>- Commercial antibodies: RRID (if possible) or supplier name, catalogue number and or/clone number<br>- Non-commercial: RRID or citation	Yes	Materials and Methods

DNA and RNA sequences	Information included in the manuscript?	In which section is the information available? (Reagents and Tools Table, Materials and Methods, Figures, Data Availability Section)
**Short novel DNA or RNA including primers, probes**: provide the sequences.	Not Applicable	

Cell materials	Information included in the manuscript?	In which section is the information available? (Reagents and Tools Table, Materials and Methods, Figures, Data Availability Section)
**Cell lines:** Provide species information, strain. Provide accession number in repository **OR** supplier name, catalog number, clone number, and/**OR** RRID.	Yes	Materials and Methods
**Primary cultures:** Provide species, strain, sex of origin, genetic modification status.	Not Applicable	
Report if the cell lines were recently **authenticated** (e.g., by STR profiling) and tested for mycoplasma contamination.	Yes	Materials and Methods

Experimental animals	Information included in the manuscript?	In which section is the information available? (Reagents and Tools Table, Materials and Methods, Figures, Data Availability Section)
**Laboratory animals or Model organisms:** Provide species, strain, sex, age, genetic modification status. Provide accession number in repository **OR** supplier name, catalog number, clone number, **OR** RRID.	Yes	Materials and Methods
**Animal observed in or captured from the field:** Provide species, sex, and age where possible.	Not Applicable	
Please detail **housing and husbandry conditions**.	Yes	Materials and Methods

Plants and microbes	Information included in the manuscript?	In which section is the information available? (Reagents and Tools Table, Materials and Methods, Figures, Data Availability Section)
**Plants:** provide species and strain, ecotype and cultivar where relevant, unique accession number if available, and source (including location for collected wild specimens).	Not Applicable	
**Microbes:** provide species and strain, unique accession number if available, and source.	Not Applicable	

Human research participants	Information included in the manuscript?	In which section is the information available? (Reagents and Tools Table, Materials and Methods, Figures, Data Availability Section)
If collected and within the bounds of privacy constraints report on age, sex and gender or ethnicity for all study participants.	Not Applicable	

Core facilities	Information included in the manuscript?	In which section is the information available? (Reagents and Tools Table, Materials and Methods, Figures, Data Availability Section)
If your work benefited from core facilities, was their service mentioned in the acknowledgments section?	Yes	Acknowledgements

Design

Study protocol	Information included in the manuscript?	In which section is the information available? (Reagents and Tools Table, Materials and Methods, Figures, Data Availability Section)
If study protocol has been **pre-registered, provide DOI in the manuscript**. For clinical trials, provide the trial registration number **OR** cite DOI.	Not Applicable	
Report the **clinical trial registration number** (at ClinicalTrials.gov or equivalent), where applicable.	Not Applicable	

Laboratory protocol	Information included in the manuscript?	In which section is the information available? (Reagents and Tools Table, Materials and Methods, Figures, Data Availability Section)
Provide DOI OR other citation details if **external detailed step-by-step protocols** are available.	Not Applicable	

Experimental study design and statistics	Information included in the manuscript?	In which section is the information available? (Reagents and Tools Table, Materials and Methods, Figures, Data Availability Section)
Include a statement about **sample size** estimate even if no statistical methods were used.	Not Applicable	
Were any steps taken to minimize the effects of subjective bias when allocating animals/samples to treatment (e.g. **randomization procedure**)? If yes, have they been described?	Not Applicable	
Include a statement about **blinding** even if no blinding was done.	Yes	Materials and Methods
Describe **inclusion/exclusion criteria** if samples or animals were excluded from the analysis. Were the criteria pre-established? If sample or data points were omitted from analysis, report if this was due to attrition or intentional exclusion and provide justification.	Not Applicable	
For every figure, are **statistical tests** justified as appropriate? Do the data meet the assumptions of the tests (e.g., normal distribution)? Describe any methods used to assess it. Is there an estimate of variation within each group of data? Is the variance similar between the groups that are being statistically compared?	Yes	Figure Legend and Methods

Sample definition and in-laboratory replication	Information included in the manuscript?	In which section is the information available? (Reagents and Tools Table, Materials and Methods, Figures, Data Availability Section)
In the figure legends: state number of times the experiment was **replicated** in laboratory.	Yes	
In the figure legends: define whether data describe **technical or biological replicates**.	Yes	

Ethics

Ethics	Information included in the manuscript?	In which section is the information available? (Reagents and Tools Table, Materials and Methods, Figures, Data Availability Section)
Studies involving **human participants**: State details of **authority granting ethics approval** (IRB or equivalent committee(s), provide reference number for approval.	Not Applicable	
Studies involving **human participants**: Include a statement confirming that **informed consent** was obtained from all subjects and that the experiments conformed to the principles set out in the WMA Declaration of Helsinki and the Department of Health and Human Services Belmont Report.	Not Applicable	
Studies involving **human participants:** For publication of **patient photos**, include a statement confirming that consent to publish was obtained.	Not Applicable	
Studies involving experimental **animals**: State details of **authority granting ethics approval** (IRB or equivalent committee(s), provide reference number for approval. Include a statement of compliance with ethical regulations.	Yes	Materials and Methods
Studies involving **specimen and field samples:** State if relevant **permits** obtained, provide details of authority approving study; if none were required, explain why.	Not Applicable	

Dual Use Research of Concern (DURC)	Information included in the manuscript?	In which section is the information available? (Reagents and Tools Table, Materials and Methods, Figures, Data Availability Section)
Could your study fall under dual use research restrictions? Please check biosecurity documents and list of **select agents and toxins** (CDC): https://www.selectagents.gov/sat/list.htm	Not Applicable	
If you used a select agent, is the security level of the lab appropriate and reported in the manuscript?	Not Applicable	
If a study is subject to dual use research of concern regulations, is the name of the **authority granting approval and reference number** for the regulatory approval provided in the manuscript?	Not Applicable	

Reporting

The MDAR framework recommends adoption of discipline-specific guidelines, established and endorsed through community initiatives. Journals have their own policy about requiring specific guidelines and recommendations to complement MDAR.

Adherence to community standards	Information included in the manuscript?	In which section is the information available? (Reagents and Tools Table, Materials and Methods, Figures, Data Availability Section)
State if relevant guidelines or checklists (e.g., **ICMJE, MIBBI, ARRIVE, PRISMA**) have been followed or provided.	Not Applicable	
For **tumor marker prognostic studies,** we recommend that you follow the **REMARK** reporting guidelines (see link list at top right). See author guidelines, under 'Reporting Guidelines'. Please confirm you have followed these guidelines.	Not Applicable	
For **phase II and III randomized controlled trials**, please refer to the **CONSORT** flow diagram (see link list at top right) and submit the CONSORT checklist (see link list at top right) with your submission. See author guidelines, under 'Reporting Guidelines'. Please confirm you have submitted this list.	Not Applicable	

Data Availability

Data availability	Information included in the manuscript?	In which section is the information available? (Reagents and Tools Table, Materials and Methods, Figures, Data Availability Section)
Have **primary datasets** been deposited according to the journal's guidelines (see 'Data Deposition' section) and the respective accession numbers provided in the Data Availability Section?	Yes	Data Availability section
Were **human clinical and genomic datasets** deposited in a public access-controlled repository in accordance to ethical obligations to the patients and to the applicable consent agreement?	Not Applicable	
Are **computational models** that are central and integral to a study available without restrictions in a machine-readable form? Were the relevant accession numbers or links provided?	Not Applicable	
If publicly available data were reused, provide the respective **data citations in the reference list**.	Not Applicable	
